# Total glucosides of paeony inhibits the differentiation and migration of intestinal mucosal immune Th17 cells in Sjogren’s syndrome mice

**DOI:** 10.3389/fimmu.2026.1742485

**Published:** 2026-10-13

**Authors:** Ge Liu, Jing Liu, Xingyu Yan, Teng Chu, Yue Wu, Weirong Fang

**Affiliations:** School of Basic Medicine and Clinical Pharmacy, China Pharmaceutical University, Nanjing, China

**Keywords:** Sjogren's syndrome, total glucosides of paeony, intestinal mucosal immunity, Th17 cells, dendritic cells

## Abstract

**Background:**

Sjogren’s syndrome (SS) is marked by exocrine-gland infiltration and Th17-driven inflammation; low-bioavailability Total glucosides of paeony (TGP) shows efficacy yet unclear mechanisms.

**Purpose:**

To investigate the role of intestinal mucosal immunity in SS pathogenesis and the effect of TGP on the differentiation and migration of Th17 in intestinal mucosal immunity.

**Methods:**

SS was induced in Peyer’s patches (PPs)/mesenteric lymph nodes (MLNs)-deficient mice. Saliva/tear flow, gland histopathology, Th17-related cytokine mRNA and blood Th17 were assessed. Established SS mice were treated with 6-week TGP. Flow cytometry/RT-qPCR evaluated CD4^+^ T cell proliferation, dendritic cell (DC) activation, and Th17 differentiation, migration and homing in intestinal mucosal immunity, blood, exocrine glands. Immunofluorescence observed the migration of *in vitro*-polarized, CFSE-labeled Th17 cells to the submandibular glands (SMG). *In vitro*, naive CD4^+^ T cells from PPs/MLNs were cultured with cytokines or LPS-activated bone marrow DCs (BMDCs) to induce Th17 differentiation, with TGP incubated. CD4^+^ T cell proliferation, Th17 proportion and cytokine mRNA was determined.

**Results:**

PPs- or MLNs-deficient SS mice showed no significant increase in SS manifestations or Th17 markers. *In vivo*, TGP inhibited CD4^+^ T cell proliferation, Th17 differentiation and DC activation/migration, blocking Th17 gland entry and gut homing by down-regulating CCR6/CCL20, CCR9/CCL25 and α4β7/MAdCAM-1 axis. Immunofluorescence results also clearly demonstrated that TGP significantly inhibited the migration of intestinal mucosa-derived Th17 cells to the SMG. *In vitro*, TGP left BMDC activation intact but sharply suppressed BMDC-driven Th17 differentiation by reducing proliferation and Th17 cytokines.

**Conclusion:**

TGP attenuates SS inflammation by inhibiting Th17 differentiation and migration from intestinal mucosal immunity to exocrine glands and their homing to intestinal mucosa.

## 1 Introduction

Sjogren’s syndrome (SS) is a chronic inflammatory autoimmune disorder, characterized by compromised exocrine gland functionality, specifically affecting the salivary and lacrimal glands, leading to symptoms of xerostomia (dry mouth) and xerophthalmia (dry eyes) (1). The main pathological features of SS include the presence of autoantibodies in the blood and lymphocyte infiltration in the exocrine glands (2). Predominantly, the infiltrating cells consist of CD4^+^ T cells, whose activation triggers epithelial cell death and subsequent destruction of the exocrine gland tissues (3). CD4^+^ T cells can be activated to further differentiate into different helper T (Th) cell subsets, such as Th1, Th2, Th17, and T. (Treg) cells. Of these, Th17 cells are mainly found in the mucosal region and have been linked to a variety of autoimmune diseases including SS (4–6). During the early phase of SS, Th17 cells secrete pro-inflammatory cytokines such as IL-17A and act in synergy with various innate immune cells to initiate and amplify the inflammatory response; as the disease progresses into its later stages, Th17 cells contribute to the maintenance of chronic inflammation by promoting the recruitment of B cells and the formation of germinal centers (5, 7).

The intestinal mucosal immune system is a complex network of gut-associated lymphoid tissue (GALT), which is mainly composed of mesenteric lymph nodes (MLNs), Peyer’s patches (PPs) and lamina propria (LP), etc (8). Th17 cells predominantly reside in the gut in a microbiota-dependent manner. Under normal physiological conditions, they serve as a primary defense mechanism against pathogens, contributing to the maintenance of epithelial barrier integrity; under abnormal conditions such as autoimmune diseases, the internal environment of the body changes. Antigens in the intestinal lumen are captured in parts such as the PPs (9). After being presented by antigen-presenting cells (APCs) such as dendritic cells (DCs), the differentiation of self-Th17 cells can be activated. In addition, CD103^+^ DCs that capture antigens can also migrate to MLNs and stimulate the differentiation of Th17 cells within them (10). Meanwhile, Th17 cells differentiated in other intestinal-associated lymphoid tissues will also migrate to the “transit station” MLNs, and some of these Th17 cells migrate to damaged tissues, while another fraction return home to the intestinal mucosal epithelium and LP (11). This process, in turn, exacerbates the inflammatory reaction. Thus, targeting the differentiation of Th17 cells within the intestinal mucosal immune system represents a promising strategy for treating and alleviating disease symptoms.

Total glucosides of paeony (TGP), a mixture extracted from the dried roots of *Paeonia lactiflora* Pall, primarily consists of Paeoniflorin (PF). In recent years, the role of TGP in regulating immune responses has been increasingly exploited in the treatment of various autoimmune diseases (12, 13). Our previous studies demonstrated that TGP could alleviate SS-like symptoms in diseased mice and significantly decrease the proportion of Th17 cells in both peripheral blood and MLNs of SS and psoriasis mouse models (14–16). Additionally, in rats with SS, administration of TGP resulted in low plasma concentration of PF, with limited distribution in submandibular glands (SMG), lacrimal glands (LG), spleen and MLNs, but notable enrichment in the PPs (**Supplementary Figure 1**). Therefore, the mechanism of TGP *in vivo* may be related to the intestinal mucosal immune system.

In this study, we first established a mouse model of SS with targeted depletion of PPs or MLNs to investigate the intrinsic relationship between intestinal mucosal immunity and SS pathogenesis. Subsequently, an antigen-induced SS model was developed in C57BL/6J mice. The effect of TGP on the proliferation of intestinal mucosal CD4^+^ T cells, activation of DCs, differentiation of Th17 cells, and the post-differentiation migration and homing of these cells was systematically evaluated following a 6-week administration of TGP. Furthermore, naive CD4^+^ T cells were isolated from PPs and MLNs, and co-cultured with DCs or IL-6, IL-23, IL-1β, and TGF-β to delineate the molecular mechanisms underlying the inhibitory effect of TGP on Th17 cell differentiation within the intestinal mucosal immune system, thereby elucidating the pathways through which TGP mitigates inflammation in exocrine glands and the intestinal mucosa.

## 2 Materials and methods

### 2.1 Mice

Female C57BL/6J mice (18 -22 g) were purchased from Jiangsu Huachuang Sino medicine technology Co., Ltd. (Taizhou, China). All mice were accommodated in the animal facility at China Pharmaceutical University with the following housing condition under a 12-hour light-dark cycle: room temperature: 25 ± 2 °C, humidity: 40 - 70%. All animal procedures were reviewed and approved by the University’s guidelines for the care and use of laboratory animals under the approval number 2020-02-005. All experiments involving anesthesia used isoflurane (RWD) as the anesthetic. According to body weight, C57BL/6J mice were randomly divided into six groups (Sham, Model, MLN-deficient, MLN-deficient Model, PP-deficient, and PP-deficient Model) or five groups (Sham, Model, PF (300 mg/kg), and TGP (720, 360 mg/kg)).

### 2.2 Drug preparation and administration

TGP capsules (catalog number: H20055058) were acquired from Ningbo Liwah Pharmaceutical Co., Ltd (Ningbo, China). PF (catalog number: ST007001) was acquired from Shanghai Stende Standard Technical Service Co., Ltd (Shanghai, China). All test drugs were prepared with a solution of 0.5% Carboxymethylcellulose Sodium (CMC-Na), sourced from Sinopharm Chemical Reagent Co., Ltd (Shanghai, China). The administration period was 6 weeks, once daily, and the Sham and Model groups were given a corresponding volume of 0.5% CMC-Na.

The TGP dosage of mice was calculated from human dosage according to body weight ratio (The average body weight of human is 60 kg, and human dosage is 1800 mg/day).

The middle dosage of TGP in mice: 1800 mg/day ÷ 60 kg × 12 times = 360 mg/kg/day.

The high dosage of TGP in mice: 360 mg/kg/day × 2 times = 720 mg/kg/day.

### 2.3 Preparation of PP-deficient mice

Mice at 14 days post-mating were intravenously injected with 2 mg of anti-IL-7Rα monoclonal antibody (Bio X Cell) (17). The successful setup of the model was confirmed by subjecting the offspring mice to an abdominal incision along the midline to visually inspect for the presence of PPs. Mice that successfully underwent the procedure were fed normally until 8 weeks of age for the subsequent experiments.

### 2.4 Preparation of MLN-deficient mice

Three-week-old C57BL/6J mice were selected and immobilized in a supine position. The abdominal region was sterilized with alcohol-soaked cotton balls, followed by a surgical incision to expose the abdominal cavity. Within the mesentery, multiple grayish-white, oval-shaped tissues were observed, which were not firmly attached to the surrounding structures and were identified as MLNs (18). Once all MLNs had been meticulously isolated, the abdomen of the mice was sutured and they were normally reared until 8 weeks of age for the subsequent experiments.

### 2.5 Establishment of SS mouse model

As previously mentioned, SS modeling was performed on C57BL/6J mice by immune induction (16). Specifically, after the mice were sacrificed by cervical dislocation, the bilateral SMG was removed under aseptic conditions. After weighing, an appropriate amount of phosphate-buffered saline (PBS) was added to prepare 10% tissue homogenate, and the supernatant was collected after centrifugation at 3000 rpm at 4 °C for 10 min. After the protein concentration was determined by BCA method, an appropriate amount of PBS and complete Freund’s adjuvant (Sigma) were added to prepare an emulsion with a final concentration of 2 mg/ml. On days 0 and 7, each mouse was injected subcutaneously with 0.1 ml of this solution, and then immunized again on day 14 with an emulsion containing 1 mg/ml of antigen mixed with incomplete Freund’s adjuvant (Sigma).

### 2.6 Naive CD4^+^ T cell isolation and Th17 cell differentiation

The PPs and MLNs of mice were isolated and placed in a 75 μm strainer for grinding, during which a suitable amount of PBS was added to prepare a single cell suspension. After centrifugation at 1500 rpm for 10 min, the precipitate was obtained and naive CD4^+^ T cells were purified adhering to the instructions of the Mouse CD4^+^ T Cell Isolation Kit (Vazyme). The differentiation of naive CD4^+^ T cells into Th17 subsets was performed following previously established protocols (14). Briefly, naive CD4^+^ T cells were cultured for 72 h in RPMI-1640 medium on plates with anti-CD3 mAb (2 μg/ml, eBioscience), in conjunction with anti-CD28 mAb (2 μg/ml), IL-2 (10 ng/ml), TGF-β (5 ng/ml), IL-1β (20 ng/ml), IL-6 (20 ng/ml), and IL-23 (20 ng/ml) from eBioscience, R&D, and PeproTech.

### 2.7 Acquisition and maturation of bone marrow derived dendritic cells (BMDCs)

Bone marrow cells were collected from the femurs and tibiae of mice. After being passed through cell strainers, the cells were cultured in RPMI-1640 medium at a density of 10^6^ cells/ml, with the addition of 20 ng/ml GM-CSF (PeproTech) and 10 ng/ml IL-4 (PeproTech). The culture medium was refreshed on an alternate-day basis, and on the sixth day, both suspended and semi-adherent cells were carefully harvested for subsequent re-plating and therapeutic administration. Finally, lipopolysaccharide (LPS, Biosharp) was added on day 7 for 48 h to stimulate cell activation.

### 2.8 Saliva flow measurement

The sterile cotton ball of predetermined mass was placed under the mouse’s tongue, and the saliva-containing cotton ball was taken out and weighed after 5 minutes to determine the saliva flow expressed in mg/min (16).

### 2.9 Tear flow measurement

The phenol red thread was positioned on the inner 1/3 of the eyelid conjunctival surface of the lower eyelid of the mouse and removed 1 min later. The length of the discolored phenol red thread was measured using a vernier caliper to determine the tear flow rate, expressed in mm/min.

### 2.10 H&E staining of SMG and LG

The SMG and LG of the mice were removed, washed with PBS, and then fixed in 4% paraformaldehyde. H&E staining was conducted to evaluate the severity of inflammation in SMG and LG. The scoring criteria were as follows (19): 0, no lymphocytic infiltration; 1, mild scattered lymphocytic infiltration; 2, moderate lymphocytic infiltration (less than one infiltration focus per 4 mm^2^); 3, one infiltration focus per 4mm^2^; 4, more than one infiltration focus per 4mm^2^.

### 2.11 Preparation of single-cell suspension

Preparation of lymphocyte suspension from lymph nodes: Lymph nodes in different parts of mice were collected under sterile conditions and placed in a 75 μm strainer. While grinding, PBS was added to obtain a single-cell suspension of mouse lymph nodes.

Preparation of lymphocyte suspension from peripheral blood: After collecting mouse heart blood, the lymphocyte ring was isolated using a Mouse peripheral blood lymphocyte isolation kit (TBD science). The lymphocyte precipitate was obtained by centrifugation at 250 g for 10 min after washing with a cleaning solution, and the single-cell suspension was prepared by adding buffer solution and blowing.

Preparation of lymphocyte suspension from SMG (20): The SMG of mice was obtained and cleaned by removing blood vessels, nerves, and connective tissue. After incubation and digestion with 0.2% type II collagenase (Solarbio) at 37 °C for 1 h, lymphocyte rings were separated from the obtained cell suspension with Percoll (Solarbio), and the lymphocyte precipitate was obtained by centrifugation at 250 g for 10 min after PBS cleaning. The single-cell suspension was prepared by adding buffer and blowing.

Preparation of lymphocyte suspension from small intestinal epithelial cells and LP: The small intestine was removed under sterile conditions and placed in pre-cooled PBS, the outer surface fat was stripped and the PPs were removed. The small intestine was dissected longitudinally and washed, transferred to 1 mM Dithiothreitol (DTT) solution (Solarbio) at 37 °C and shaken at 220 rpm for 10 min, and then transferred to 30 mM Ethylenediaminetetraacetic acid (EDTA) solution (Beyotime) at 37 °C and shaken for 20 min. The collected liquid was centrifuged at 2000 rpm for 10 min to precipitate epithelial lymphocytes. The above intestinal fragments were washed with RPMI-1640 medium containing 5% fetal bovine serum (FBS) and digested with RPMI-1640 digestive solution (containing 10% FBS, 150 µg/ml DNase I (Solarbio), and 100 U/ml collagenase VIII (Sigma)) on a shaking table for 40 min. The digested cell suspension was filtered and centrifuged to obtain LP cells. The resulting precipitates were separated by Percoll, washed with PBS, and centrifuged to obtain the lymphocyte precipitates. Finally, buffer was added and blown to prepare a single-cell suspension.

### 2.12 Flow cytometry

For the measurement of IL-17A^+^ Th17 cells, Phorbol 12-Myristate 13 Acetate (PMA, Fcmacs), Brefeldin-A (BFA, Fcmacs) and Ionomycin (Fcmacs) were added into the above obtained lymphocyte suspension, and then placed in an incubator at 37 °C for 6 h. Cell surface antigens were stained with the following fluorochrome-conjugated antibodies from BioLegend and eBioscience: APC-anti-mouse CD103, BV421-anti-mouse CD45, FITC-anti-mouse CD11c, FITC-anti-mouse CD4, PE-anti-mouse MHC Class II, BV605-anti-mouse CD80, APC-anti-mouse CD86, eFluor™ 450-anti-mouse CCR9, PE-anti-mouse CCR6, and PE-anti-mouse α4β7. After breaking the membrane or nuclear membrane with a Fixation/Permeabilization Buffer Set (eBioscience) as needed, cells were incubated with the following antibodies (eBioscience) for 30 min: PE-anti-mouse RORγt, APC-anti-mouse IL-17A, and eFluor™ 660-anti-mouse Ki-67. The stained cells were subjected to analysis using Celesta flow cytometer (BD Biosciences), and the data were processed by FACSDiva and FlowJo software.

### 2.13 RT-qPCR

Total RNA was extracted from tissues (SMG, LG, MLNs, PPs, and small intestine) or cells using TRIzol reagent (Vazyme). According to the instructions of the ChamQ SYBR qPCR Master Mix (Vazyme) and the HiScript II One Step qRT-PCR SYBR Green Kit (Vazyme), RNA was reverse-transcribed into cDNA and amplified. The results were analyzed with β-actin as the internal reference gene, and the relative expression levels of the target genes were represented by calculating the 2^-ΔΔCt^ values. The primers are listed in **Supplementary Table 1**.

### 2.14 Immunofluorescence

Naive CD4^+^ T cells were isolated from mouse PPs and MLNs and polarized toward Th17 cells as described. After polarization, cells were labeled with 2.5 μM CFSE (TopScience) in RPMI-1640 medium for 20 min at room temperature in the dark. The reaction was quenched with RPMI-1640 medium containing 10% FBS, and cells were washed twice with PBS. CFSE labeled cells (2 × 10^6^) were injected intravenously into recipient mice that had been treated with 0.5% CMC-Na, PF (300 mg/kg) or TGP (720, 360 mg/kg, respectively) for 6 weeks. At 2 h post-injection, SMG were harvested, cryosectioned (8 µm), fixed in 4% paraformaldehyde for 15 min, washed with PBS, and mounted with DAPI-containing anti-fading medium (Beyotime). Images were acquired on a Leica DMi8 fluorescence microscope.

### 2.15 Statistical analysis

Data are presented as mean ± SD. Statistics to analyze differences among groups using SPSS software are indicated in figure legends. One-way analysis of variance (ANOVA) or Mann-Whitney *U* test was used to compare differences between two groups. P < 0.05 was considered statistically significant.

## 3 Results

### 3.1 Intrinsic relationship between intestinal mucosal immunity, Th17 cells and SS

To investigate the involvement of the intestinal mucosal immune system in the development and progression of SS, we employed antibody-mediated depletion or surgical resection to selectively remove PPs or MLNs in mice, followed by immune induction to establish the disease model. The results indicated that, compared with the Sham group, mice in the Model group exhibited a marked reduction in saliva and tear flow. In contrast, mice lacking MLNs demonstrated higher tear and saliva flow in both the modeled and non-modeled groups. Similarly, mice lacking PPs showed increased saliva flow in both the modeled and non-modeled groups. Notably, there were no significant differences in saliva or tear flow in mice before and after modeling with the removal of MLNs and PPs (**Figures 1a, c**). As illustrated in **Figures 1b, d**, the Model group displayed a significant augmentation of lymphocytic infiltration within the SMG and LG with respect to the Sham group. However, immune induction in mice with excised PPs or MLNs did not result in comparable pathological alterations. These observations preliminarily suggest that the absence of PPs or MLNs hinders the establishment of SS modeling, highlighting the integral role of intestinal mucosal immunity in the disease pathogenesis.

**Figure 1:**
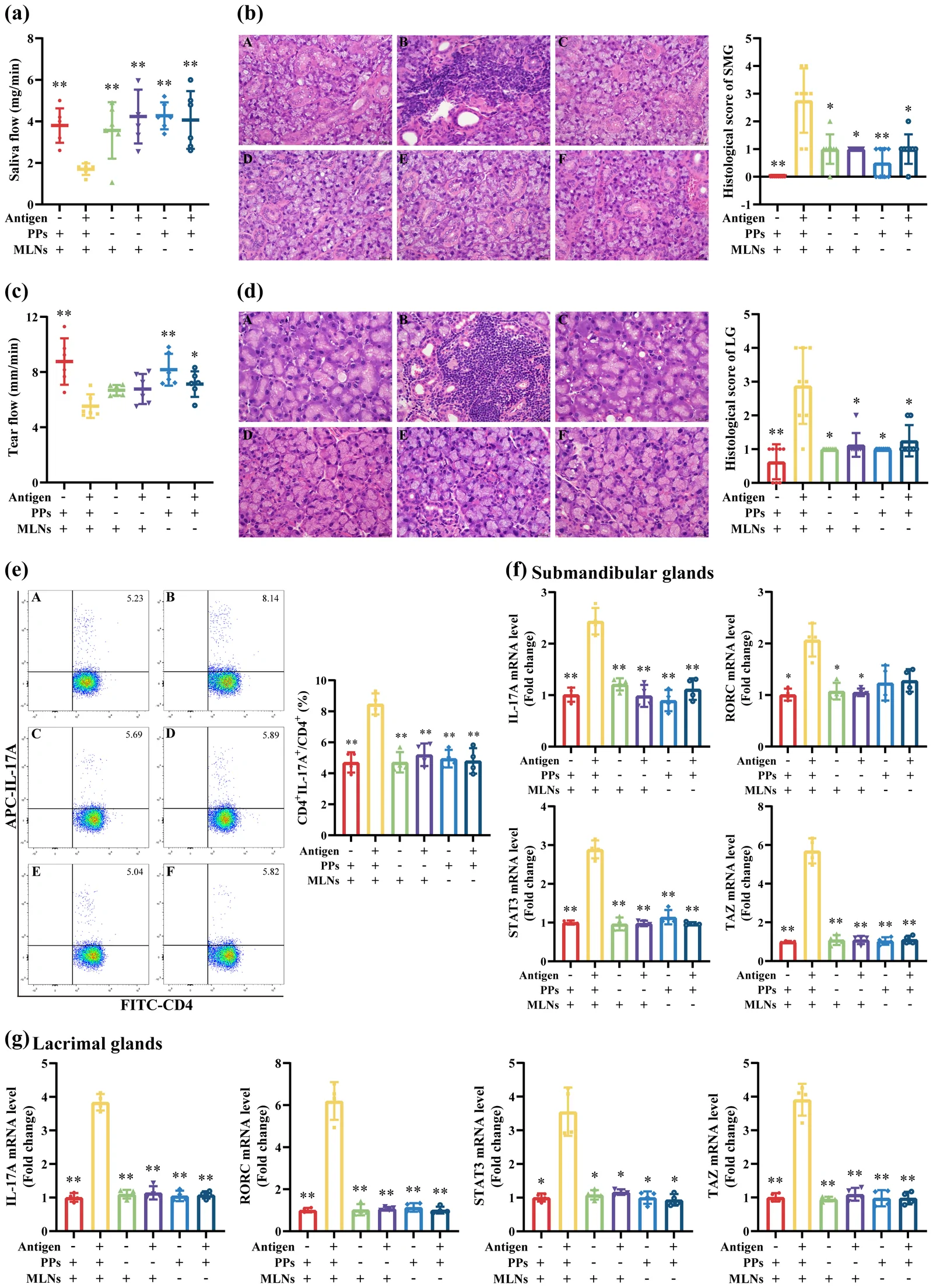
Impact of PPs or MLNs deficiency on the pathogenesis of SS. SS was induced in mice following the removal of PPs or MLNs. **(a, c)** Mean and SD of saliva **(a)** and tear **(c)** flow (*n* = 6). **(b, d)** Representative H&E staining images and histological scores (mean ± SD) for SMG **(b)** and LG **(d)**. Scale bar: 20 μm (*n* = 8). **(e)** Representative flow cytometric plots and quantitative data (mean ± SD) of IL-17A-expressing CD4^+^ T (Th17) cells in peripheral blood (*n* = 4). **(f, g)** IL-17A, RORC, STAT3 and TAZ mRNA expression levels in SMG **(f)** and LG **(g)** detected by RT-qPCR assay (mean ± SD, *n* = 4). ^*^P < 0.05, ^**^P < 0.01, compared to the model group.

To determine whether Th17 cells are implicated in this process, we assessed the proportion of Th17 cells among CD4^+^ T cells in peripheral blood from mice across all groups, as well as the mRNA expression of Th17-related cytokines (IL-17A, RORC, STAT3, and TAZ) in the SMG and LG. As depicted in **Figure 1e**, the Model group demonstrated a marked increase in the proportion of Th17 cells among CD4^+^ T cells in peripheral blood compared to the Sham group. Conversely, mice lacking PPs or MLNs, whether modeled or not, showed an obvious reduction in Th17 cell proportion as opposed to the Model group, with no significant difference observed in relation to the Sham group. Consistent trends were observed in the mRNA expression of Th17-related cytokines in the SMG and LG. Specifically, in mice with PPs and MLNs removed, whether modeled or not, the mRNA expression levels of the four cytokines were reduced in contrast to those in the Model group, but remained similar to those in the Sham group (**Figures 1f, g**).

### 3.2 TGP inhibited CD4^+^ T cell proliferation and Th17 cell differentiation within the intestinal mucosal immunity, peripheral blood and SMG of mice with SS

Based on the results presented in **Figure 1**, we utilized C57BL/6J mice to establish an SS model via immune induction, followed by 6 weeks of oral gavage with TGP and PF, to further explore whether TGP and PF could modulate CD4^+^ T cell proliferation and Th17 cell differentiation in the intestinal mucosal immune system of mice with SS. As shown in **Figures 2**, **3**, compared against the Sham group, mice in the Model group demonstrated marked increases in CD4^+^ T cell proliferation within PPs, MLNs, and the small intestinal epithelium and LP, as well as a significant elevation in the proportion of Th17 cells among CD4^+^ T cells. On the contrary, treatment with TGP and PF obviously attenuated CD4^+^ T cell proliferation in these tissues and notably inhibited Th17 cell differentiation in the small intestinal LP and MLNs. Additionally, TGP administration effectively suppressed Th17 cell differentiation in PPs. RT-qPCR also corroborated these findings. Compared to the Sham group, the mRNA expression levels of Th17 cell differentiation-related factors (IL-17A, RORC, STAT3 and TAZ) in PPs, small intestine and MLNs of mice in the Model group were significantly increased; whereas, following 6 weeks of TGP administration, the mRNA expression of the aforementioned four factors in the aforementioned tissues was markedly inhibited (**Supplementary Figure 2**).

**Figure 2:**
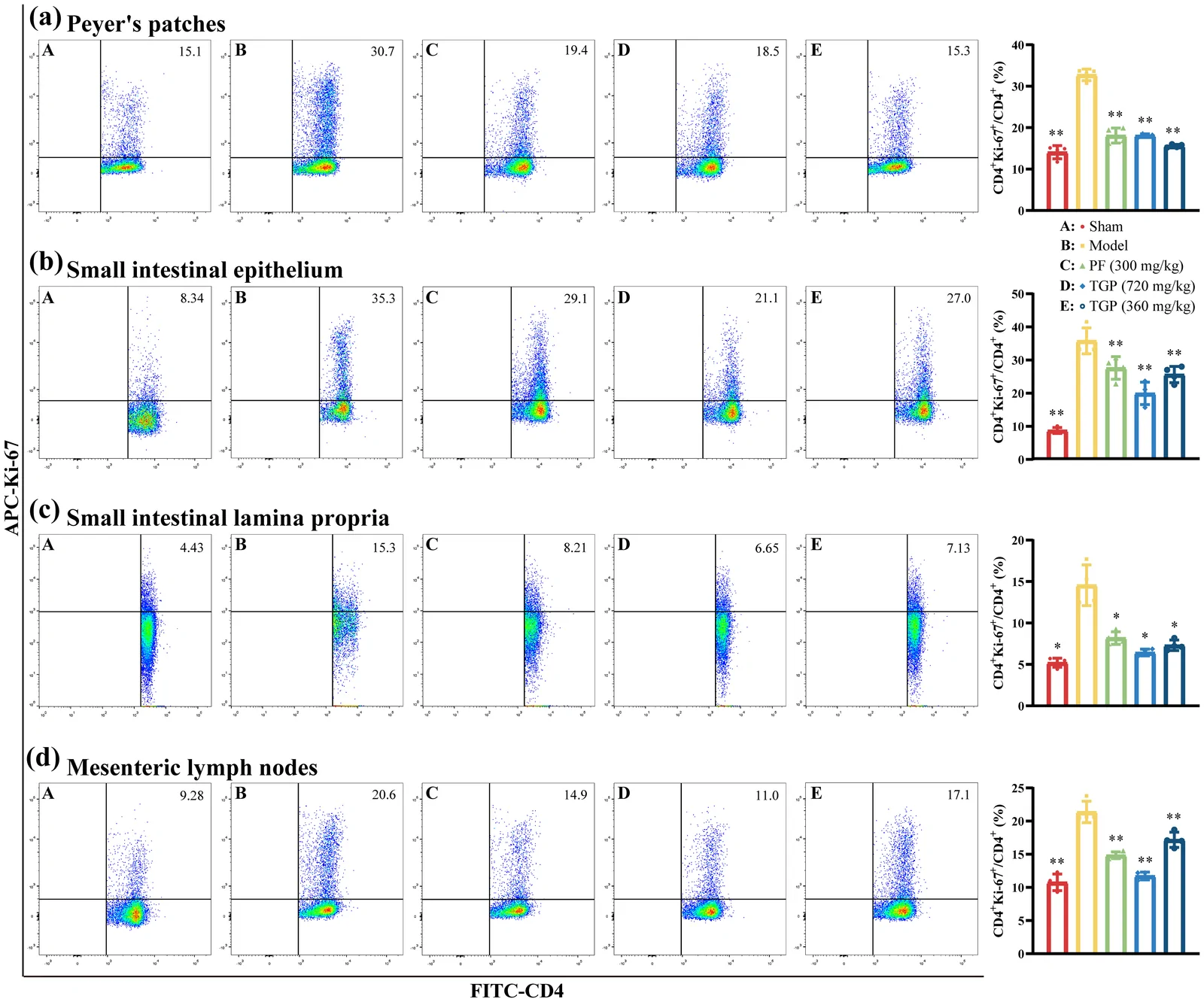
TGP inhibits the proliferation of intestinal mucosal immune CD4^+^ T cells in SS mice. SS mice were treated with vehicle (0.5% CMC-Na), PF (300 mg/kg), or TGP (720, 360 mg/kg) for 6 weeks via intragastric gavage. The proportions of CD4^+^ Ki-67^+^ T cells in CD4^+^ T cells in PPs, small intestinal epithelium, small intestinal LP, and MLNs were analyzed by flow cytometry. Representative flow cytometric plots and quantitative data (mean ± SD) of Ki-67-expressing CD4^+^ T cells in PPs **(a)**, small intestinal epithelium **(b)**, small intestinal LP **(c)**, and MLNs **(d)** of mice. ^*^P < 0.05, ^**^P < 0.01, compared to the model group. *n* = 4 mice/group.

**Figure 3:**
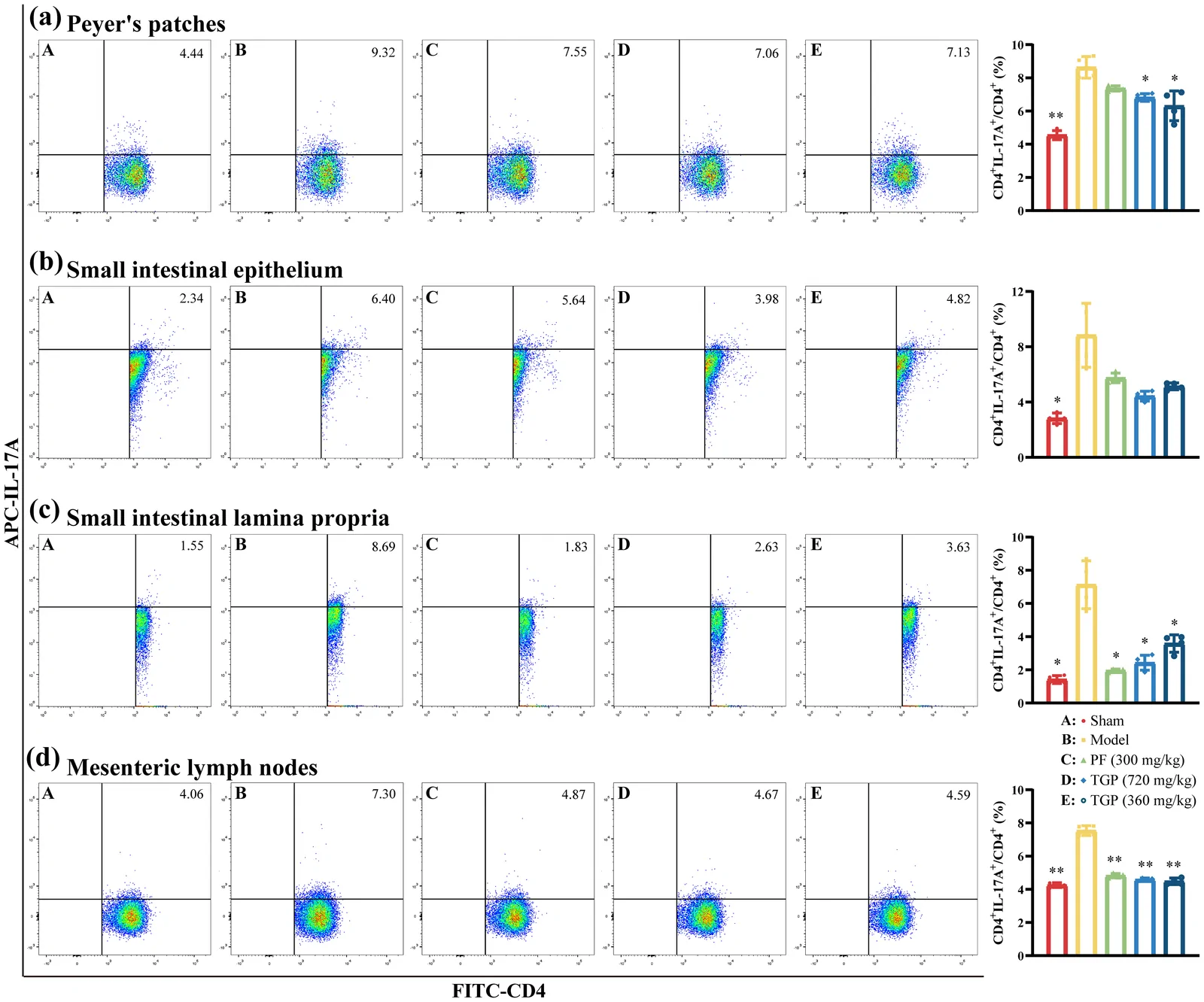
TGP suppresses the differentiation of intestinal mucosal immune CD4^+^ T cells into Th17 cells in SS mice. SS mice were treated with vehicle (0.5% CMC-Na), PF (300 mg/kg), or TGP (720, 360 mg/kg) for 6 weeks via intragastric gavage. The proportions of CD4^+^ IL-17A^+^ T cells in CD4^+^ T cells in PPs, small intestinal epithelium, small intestinal LP, and MLNs were analyzed by flow cytometry. Representative flow cytometric plots and quantitative data (mean ± SD) of IL-17A-expressing CD4^+^ T cells in PPs **(a)**, small intestinal epithelium **(b)**, small intestinal LP **(c)**, and MLNs **(d)** of mice. ^*^P < 0.05, ^**^P < 0.01, compared to the model group. *n* = 4 mice/group.

Furthermore, we also assessed CD4^+^ T cell proliferation and Th17 cell proportion in peripheral blood, SMG, and submandibular lymph nodes, finding that TGP remarkably curtailed CD4^+^ T cell proliferation and down-regulated the proportion of Th17 cells among CD4^+^ T cells in these tissues (**Figure 4**). Given the low plasma concentration of PF, the major component of TGP, and its limited distribution in exocrine glands, the results further indicate that the reduction of Th17 cell and other lymphocyte infiltration in exocrine glands and systemically by TGP is related to its ability to inhibit the differentiation of intestinal mucosal immune Th17 cells.

**Figure 4:**
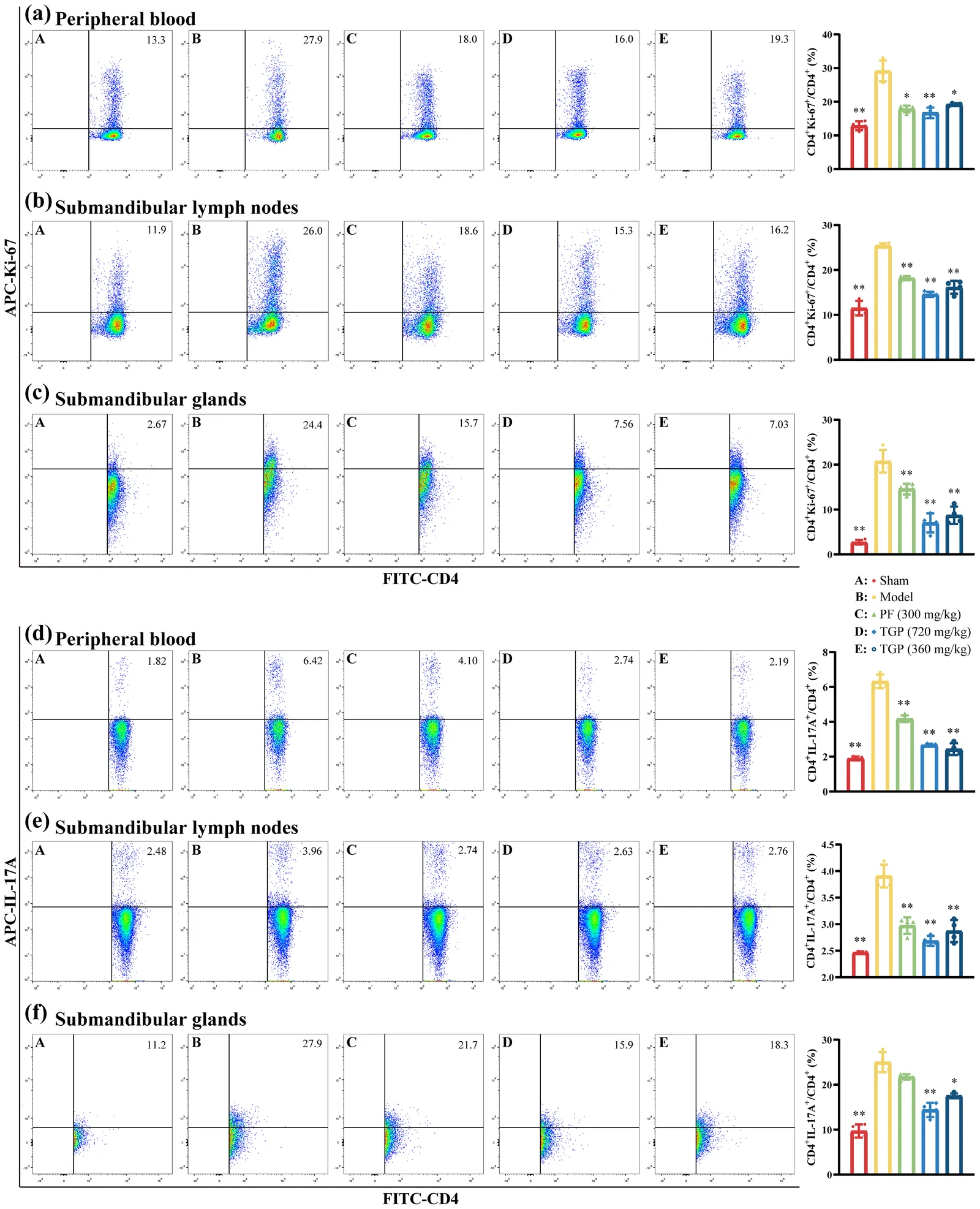
TGP inhibits CD4^+^ T cell proliferation and Th17 cell differentiation in the peripheral blood and SMG of SS mice. SS mice were treated with vehicle (0.5% CMC-Na), PF (300 mg/kg), or TGP (720, 360 mg/kg) for 6 weeks via intragastric gavage. The proportions of CD4^+^ Ki-67^+^ T cells and CD4^+^ IL-17A^+^ T cells in CD4^+^ T cells in peripheral blood, submandibular lymph nodes, and SMG were analyzed by flow cytometry. Representative flow cytometric plots and quantitative data (mean ± SD) of Ki-67- or IL-17A-expressing CD4^+^ T cells in peripheral blood **(a, d)**, submandibular lymph nodes **(b, e)**, and SMG **(c, f)** of mice. ^*^P < 0.05, ^**^P < 0.01, compared to the model group. *n* = 4 mice/group.

In the pathogenesis of certain autoimmune diseases, migratory DCs (CD103^+^ DCs) in the LP migrate to MLNs and drive Th17 cell differentiation by secreting IL-6 and other effector molecules. As shown in **Figures 5a, b**, compared with the Sham group, migratory DCs were significantly decreased in the small intestinal LP and increased in MLNs in the Model group, accompanied by up-regulated IL-6 and IL-23 mRNA expression in MLNs. At the same time, TGP and PF administration notably inhibited DC migration and effector molecule secretion. Activated DCs in damaged glands can also induce Th17 cell differentiation. As illustrated in **Figures 5c–e**, compared to Sham mice, the proportion of activated DCs (CD80^+^ CD86^+^ DCs) in the SMG and the mRNA expression of IL-6 and IL-23 in the SMG and LG of Model mice exhibited a substantial increase. TGP and PF therapy effectively suppressed the activation of DCs and IL-6/IL-23 mRNA expression in exocrine glands.

**Figure 5:**
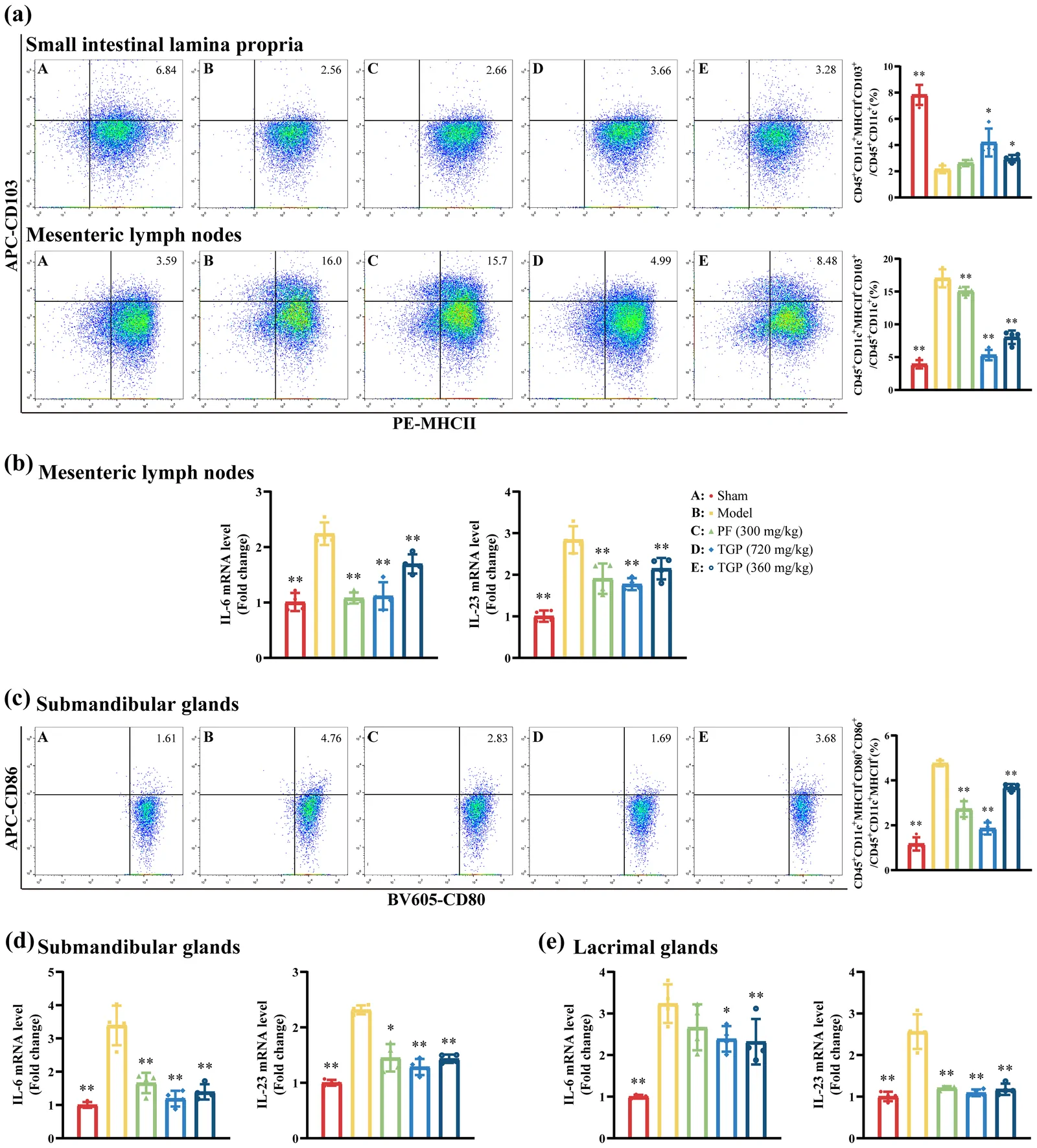
TGP suppresses the migration and activation of DCs from intestinal mucosal immunity and SMG in SS mice. SS mice were treated with vehicle (0.5% CMC-Na), PF (300 mg/kg), or TGP (720, 360 mg/kg) for 6 weeks via intragastric gavage. Representative flow cytometric plots and quantitative data of CD103^+^ MHCII^+^ DCs in small intestinal LP and MLNs **(a)**, and CD80^+^ CD86^+^ DCs in SMG **(c)** of mice were shown. The mRNA expression levels of IL-6 and IL-23 in MLNs **(b)**, SMG **(d)**, and LG **(e)** were analyzed by RT-qPCR. All data are mean ± SD. ^*^P < 0.05, ^**^P < 0.01, compared to the model group. *n* = 4 mice/group.

### 3.3 TGP inhibited the migration of Th17 cells from intestinal mucosal immunity to SMG and homing to intestinal mucosa in mice with SS

Building on our previous findings, we delved deeper into how TGP influences the migration of Th17 cells in the intestinal mucosal immune system. Sphingosine 1-phosphate receptor 1 (S1PR1) is pivotal for lymphocyte migration, and Krüppel-like transcription factor 2 (KLF2) regulates its expression (21, 22). As depicted in **Figure 6a**, S1PR1 and KLF2 mRNA levels in MLNs of mice were significantly increased in Model group versus Sham group, while TGP and PF treatments substantially suppressed this up-regulation. During infection and autoimmune disorders, CCR6 interacts with its ligand CCL20 to facilitate the recruitment of Th17 cells to inflammatory sites, and the inflammatory sites can further promote this migration by releasing chemokines (23). As demonstrated in **Figure 6b**, the percentage of CCR6^+^ Th17 cells among CD4^+^ T cells in SMG was notably higher in mice of the Model group than in those of the Sham group, and was markedly reduced in all medicated groups compared with the Model group. RT-qPCR revealed that, relative to the Sham group, expression levels of CCL20 and CXCL10 in SMG and LG were significantly elevated in the Model group, and TGP and PF obviously inhibited the mRNA expression of CCL20 and CXCL10 in SMG and LG of SS mice (**Figures 6c, d**). In parallel, naive CD4^+^ T cells isolated from the PPs and MLNs of mice were polarized toward Th17 cells *in vitro*, labeled with CFSE, and then injected into the corresponding groups of mice via the tail vein. The results showed that the CFSE/DAPI ratio in the SMG was notably reduced in all treatment groups compared with the Model group, providing direct evidence that TGP inhibits the migration of intestinal mucosa-derived Th17 cells to the damaged gland tissue. (**Figure 6e**).

**Figure 6:**
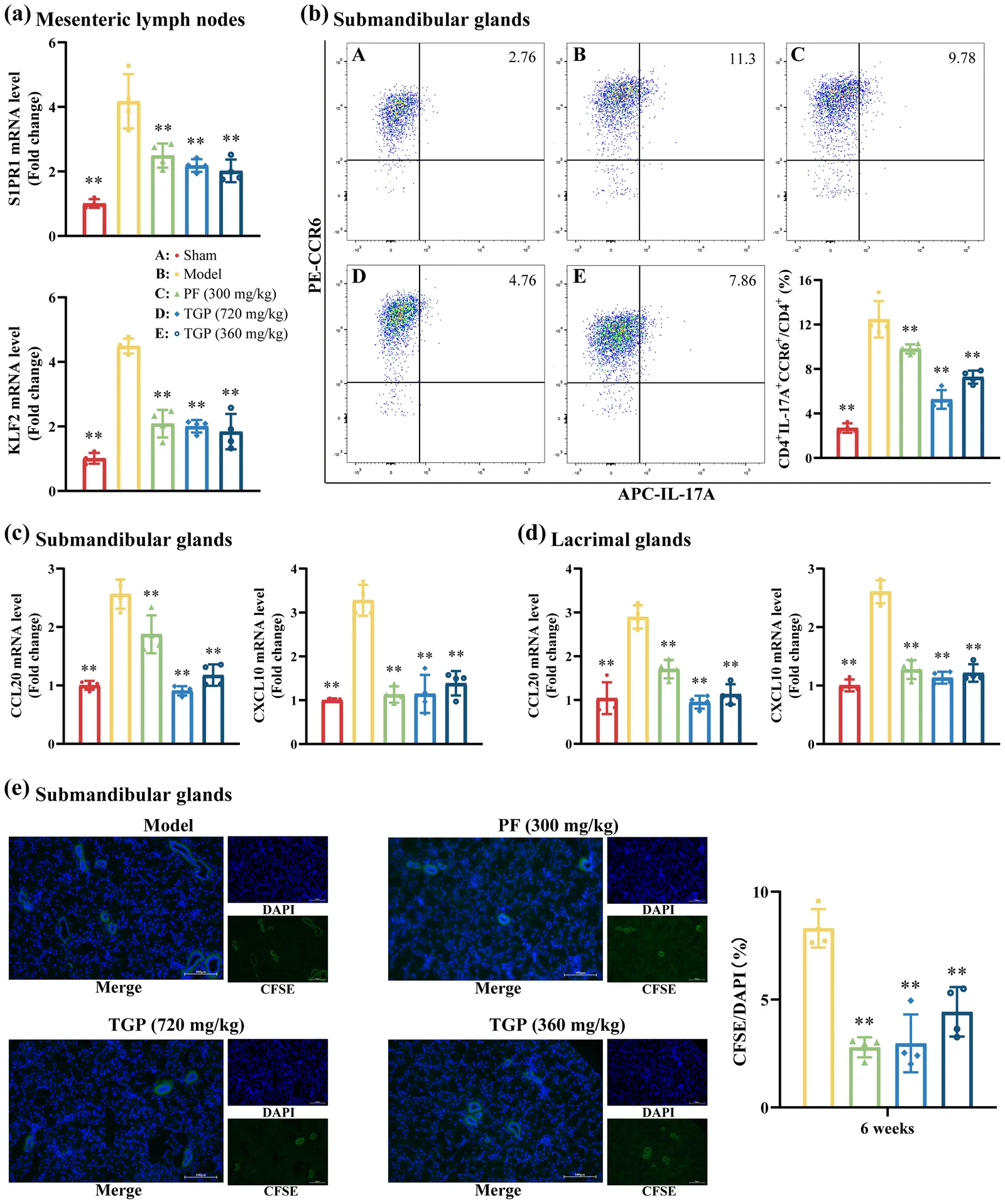
TGP suppresses the effusion and migration of intestinal mucosal immune Th17 cells to exocrine glands in SS mice. SS mice were treated with vehicle (0.5% CMC-Na), PF (300 mg/kg), or TGP (720, 360 mg/kg) for 6 weeks via intragastric gavage. **(a)** S1PR1 and KLF2 mRNA expression levels in MLNs detected by RT-qPCR assay. **(b)** Representative flow cytometric plots and quantitative data of CCR6^+^ Th17 cells in SMG. **(c, d)** CCL20 and CXCL10 mRNA expression levels in SMG **(c)** and LG **(d)** detected by RT-qPCR assay. **(e)** Immunofluorescence staining and analysis of CFSE expression in SMG. Scale bar: 100 μm. All data are mean ± SD. ^**^P < 0.01, compared to the model group. *n* = 4 mice/group.

As shown in **Figures 7a, d**, compared to the Sham group, the proportion of CCR6^+^ Th17 cells in the small intestinal epithelium and LP and the mRNA expression of CCL20 in the small intestine were notably increased in the Model group, which indicates that the CCR6/CCL20 axis can not only promote the migration of Th17 cells to the inflammatory site, but also guide their homing to the intestinal mucosa. Compared with the Model group, CCR6 expression in the small intestinal LP of all drug-treated mice was significantly reduced, along with a marked decrease in CCL20 mRNA expression in the small intestine. Some Th17 cells recirculate in the gut mucosa via the α4β7 integrin/mucosal addressin cell adhesion molecule-1 (MAdCAM-1) axis or the CCR9/CCL25 axis (24). As presented in **Figures 7b–d**, in the Model group, the proportion of CCR9^+^ Th17 cells in the small intestinal epithelium and α4β7^+^ Th17 cells in the LP among CD4^+^ T cells, as well as the CCL25 and MAdCAM-1 mRNA levels in the small intestine, were all notably higher than in the Sham group. Compared with the Model group, TGP and PF treatments resulted in a marked reduction in the expression of CCR9, α4β7, CCL25, and MAdCAM-1 in the small intestine.

**Figure 7:**
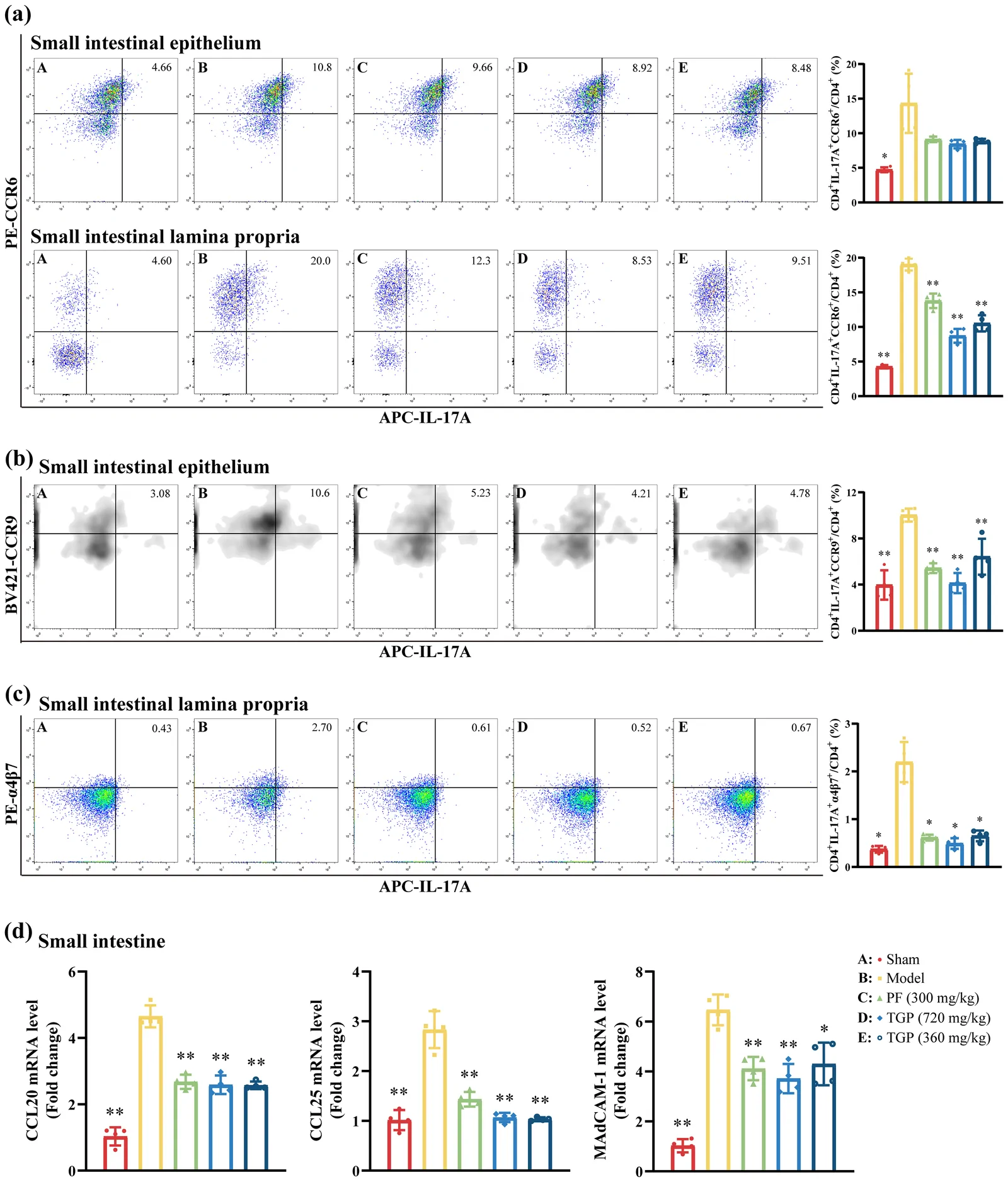
TGP suppresses intestinal mucosal immune Th17 cells homing back to intestinal mucosa in SS mice. SS mice were treated with vehicle (0.5% CMC-Na), PF (300 mg/kg), or TGP (720, 360 mg/kg) for 6 weeks via intragastric gavage. **(a–c)** Representative flow cytometric plots and quantitative data of CCR6^+^ Th17 cells in small intestinal epithelium and LP **(a)**, CCR9^+^ Th17 cells in small intestinal epithelium **(b)**, and α4β7^+^ Th17 cells in small intestinal LP **(c)**. **(d)** CCL20, CCL25 and MAdCAM-1 mRNA expression levels in small intestine detected by RT-qPCR assay. All data are mean ± SD. ^*^P < 0.05, ^**^P < 0.01, compared to the model group. *n* = 4 mice/group.

### 3.4 Effect of TGP on the differentiation of naive CD4^+^ T cells into Th17 cells and its mechanism

Finally, we further shed light on the mechanism by which TGP inhibits Th17 cell differentiation through *in vitro* experiments. Consistent with our *in vivo* findings, as shown in **Figure 8a**, CD4^+^ T cell proliferation was significantly suppressed in the TGP-treated group compared to the Vehicle group under the stimulation of cytokines IL-6, IL-23, IL-1β and TGF-β. Additionally, after three days of treatment with TGP and PF, the proportion of Th17 cells among CD4^+^ T cells and the mRNA expression of Th17-related cytokines were notably diminished (**Figures 8b, c**).

**Figure 8:**
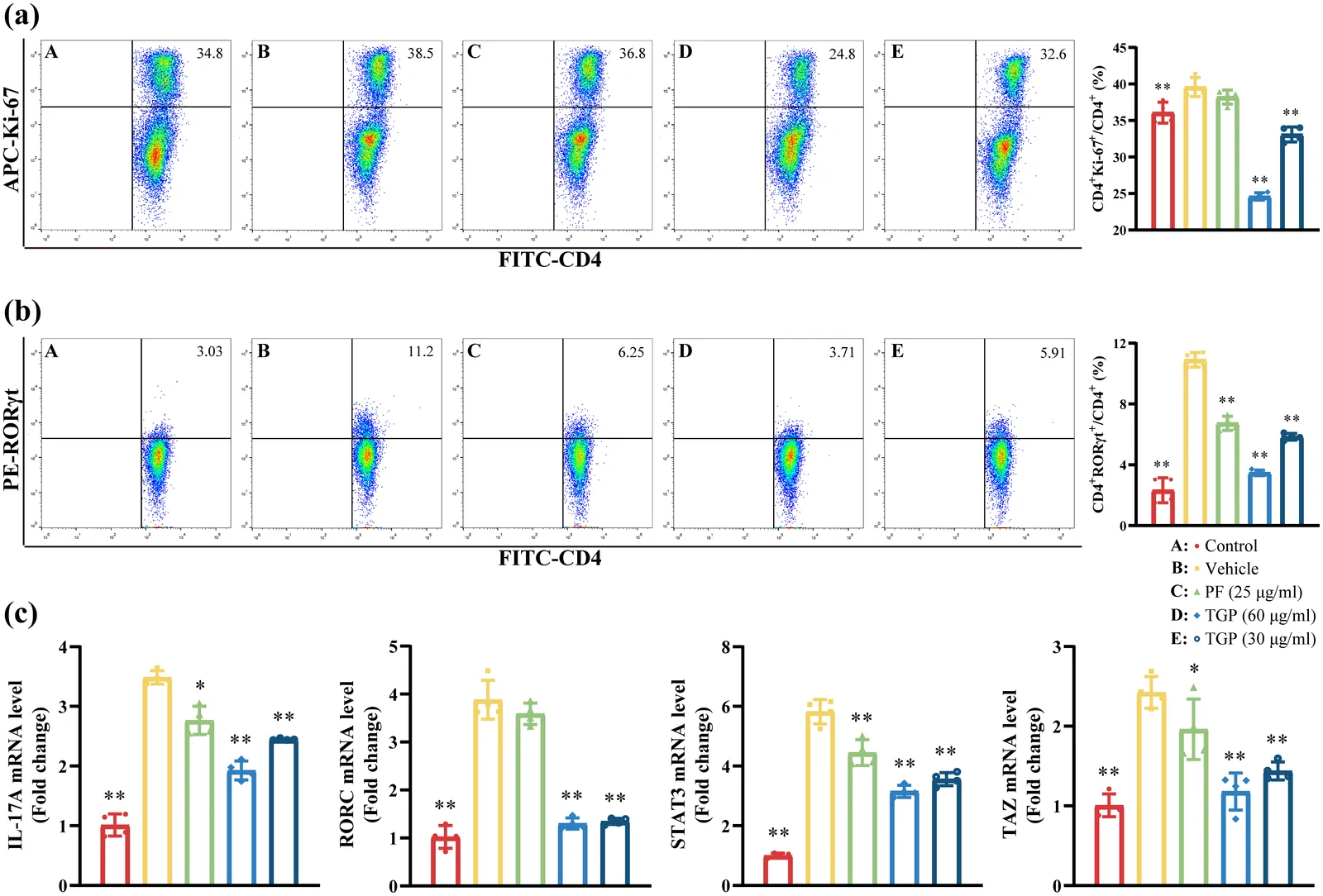
TGP inhibits the differentiation of naive CD4^+^ T cells into Th17 cells induced by TGF-β, IL-1β, IL-6, and IL-23. Naive CD4^+^ T cells were purified from mouse MLNs and PPs, differentiated in the presence of TGF-β (5 ng/ml), IL-1β (20 ng/ml), IL-6 (20 ng/ml), and IL-23 (20 ng/ml) for 72 h, and analyzed for the proliferation of naive CD4^+^ T cells and their differentiation into Th17 cells. **(a)** Representative flow cytometric plots and quantification of Ki-67-expressing CD4^+^ T cells (*n* = 4). **(b)** Representative flow cytometric plots and quantification of RORγt-expressing Th17 cells (*n* = 4). **(c)** mRNA levels of IL-17A, RORC, STAT3, and TAZ in differentially treated CD4^+^ T cells analyzed by RT-qPCR assay (*n* = 4). All data are mean ± SD. ^*^P < 0.05, ^**^P < 0.01, compared to the vehicle treatment.

As activated DCs also contribute to Th17 cell differentiation and migration, we investigated whether TGP could modulate DC activation *in vitro*. As evidenced in **Figure 9a**, although LPS markedly up-regulated the proportion of CD80^+^ CD86^+^ DCs, neither TGP nor PF inhibited LPS-induced BMDCs activation. Accordant with this finding, TGP and PF did not alter the elevated mRNA levels of IL-6, IL-23, IL-1β, or the reduced mRNA level of TGF-β in activated BMDCs, all of which serve as key factors in Th17 cell differentiation (**Figure 9b**). Building on these results, we co-cultured gut-derived naive CD4^+^ T cells with either activated or non-activated BMDCs at a ratio of 10:1 for three days, with PF and TGP administered throughout the process. As illustrated in **Figures 9c, d**, as opposed to naive CD4^+^ T cells co-cultured with non-activated BMDCs, those co-cultured with LPS-activated BMDCs presented significant proliferation and differentiation into Th17 cells. Remarkably, treatment with TGP and PF evidently attenuated these effects. Based on these observations, we hypothesized that TGP and PF primarily target Th17 cells. To test this hypothesis, we measured the mRNA expression levels of factors associated with Th17 cell differentiation. As expected, TGP and PF appreciably lowered the mRNA expression of IL-17A, RORC, STAT3, and TAZ (**Figure 9e**).

**Figure 9:**
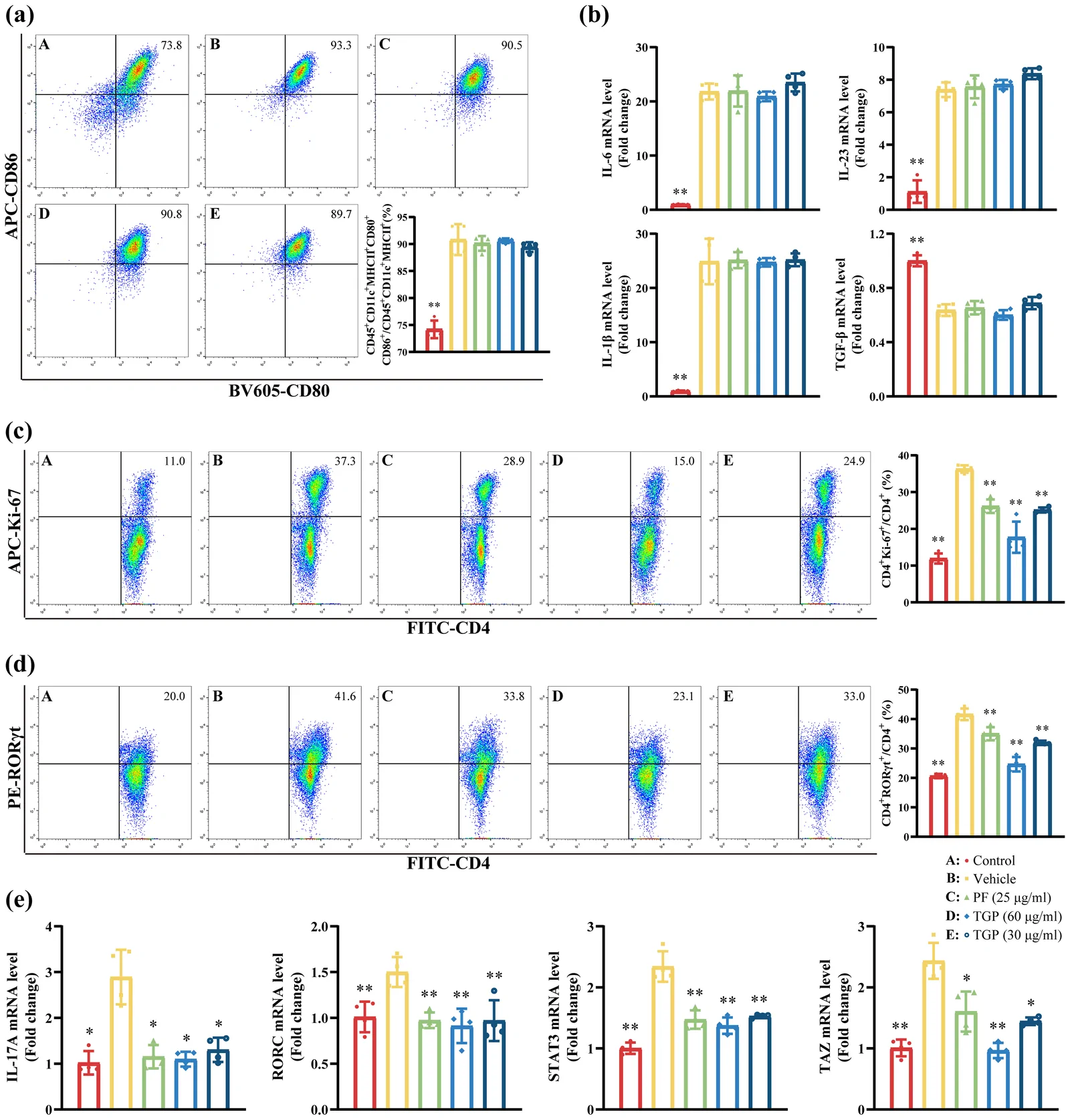
TGP inhibits the differentiation of naive CD4^+^ T cells into Th17 cells induced by activated DCs. Naive CD4^+^ T cells were purified from mouse MLNs and PPs, differentiated by co-culture with LPS-activated BMDCs at a ratio of 10:1 for 72 h and analyzed for the naive CD4^+^ T cells proliferation and their differentiation into Th17 cells. Naive CD4^+^ T cells co-cultured with non-activated BMDCs were used as Control. **(a)** Representative flow cytometric plots and quantification of activated BMDCs (CD80^+^ CD86^+^ DCs). **(b)** mRNA levels of IL-6, IL-23, IL-1β, and TGF-β in differentially treated BMDCs analyzed by RT-qPCR assay. **(c)** Representative flow cytometric plots and quantification of Ki-67-expressing CD4^+^ T cells. **(d)** Representative flow cytometric plots and quantification of RORγt-expressing Th17 cells. **(e)** mRNA levels of IL-17A, RORC, STAT3, and TAZ in differentially treated CD4^+^ T cells analyzed by RT-qPCR assay. All data are mean ± SD, *n* = 4. ^*^P < 0.05, ^**^P < 0.01, compared to the vehicle treatment.

## 4 Discussion

SS is a chronic inflammatory autoimmune disorder that predominantly targets the exocrine glands and is typified by diminished secretory function of the SMG and LG (25). Clinically, SS is characterized by dry mouth and dry eyes, as well as multisystemic manifestations stemming from involvement of other glands and extraglandular organs. The pathogenesis of SS is complex, encompassing the activation of diverse immune cells and the secretion of numerous cytokines. Among these, over-activation of T cells, particularly CD4^+^ T cells, plays a crucial role in the initiation and progression of the disease. Upon activation, CD4^+^ T cells can differentiate into distinct subsets, including Th1, Th2, Th17, and Treg cells (26). Since the seminal identification of Th17 cell infiltration in the SMG of SS patients in 2008, subsequent studies have established Th17 cells as a key mediator of inflammatory responses in the exocrine glands. TGP, an active constituent derived from the traditional Chinese medicine *Paeonia lactiflora* Pall, exhibits a spectrum of pharmacological activities, including immunomodulatory effects, with PF being its principal bioactive component. However, the bioavailability of TGP is very low. Our previous studies have also shown that after intragastric administration of TGP to SS rats, the blood concentration of PF is relatively low, and it is rarely distributed in SMG and LG, but is enriched in PPs, with trace amounts in MLNs (**Supplementary Figure 1**). On this basis, we prepared SS models by immune induction after removing PPs or MLNs from mice. The results showed that PPs- or MLNs-deficient mice exhibited markedly alleviated SS-like symptoms and a significantly reduced Th17 cell response (**Figure 1**). Therefore, both the development of SS and its treatment with TGP are strongly tied to intestinal mucosal immunity.

The intestinal mucosal immune system, principally constituted by GALT, encompasses not only PPs and MLNs, but also the intestinal epithelium and LP. These components, while functionally interrelated, operate independently to collectively form the intestinal immune barrier. Intestinal epithelial cells form the first physical barrier of the intestine, preventing pathogens from directly accessing the intestinal lumen. PPs are the main induction sites of this system, and the microfold cells (M cells) at their top can capture antigens in the intestinal lumen (9). The LP of the intestinal mucosa is scattered with a diverse array of immune cells, including T cells, B cells, macrophages, DCs, etc. MLNs can receive lymph fluid from the intestine and are the sites where immune cells gather and immune responses further develop. Under pathophysiological conditions such as autoimmune diseases, alterations in the host’s internal environment or gut microbiota can induce the activation of M cells in PPs. These M cells capture antigens, which on one hand can activate the differentiation of self-Th17 cells, and some differentiated Th17 cells will migrate to MLNs through lymphatic vessels; on the other hand, they can be passed on to the immune cells below, such as the large number of T cells, B cells, and APCs scattered in the LP of the intestinal mucosa. Among these, T and B cells can be activated and further differentiated, and APCs can also migrate to MLNs to further present antigens to naive CD4^+^ T cells and promote their activation (27). DCs are the most potent professional APCs in the body and occupy a pivotal position in the initiation, regulation, and maintenance of immune responses (28). In addition to efficiently capturing, processing, and presenting antigens, activated DCs can also effectively activate naive CD4^+^ T cells by secreting effector factors such as IL-6 and IL-23 (29). Our results showed that TGP could directly inhibit the differentiation of intestinal mucosal immune Th17 cells by reducing the proliferation of CD4^+^ T cells and the mRNA expression of key transcriptional molecules of Th17 cells in PPs, small intestinal epithelium and LP, and MLNs; at the same time, TGP can also indirectly inhibit the differentiation of intestinal mucosal immune Th17 cells by reducing the migration of DCs from the small intestinal LP to MLNs and the secretion of effector factors (**Figures 2**, **3**, **5**).

Next, we further investigated whether TGP could affect the excretion and migration of differentiated Th17 cells to damaged tissues and their homing to the intestinal mucosa. S1PR1, a G protein-coupled receptor, regulates the migration and differentiation of immune cells and plays a key role in the excretion of T cells from secondary lymphoid organs (such as lymph nodes) into the blood and lymphatic vessels (21). KLF2 is a transcription factor that can directly bind to the promoter region of S1PR1 gene and regulate its expression by activating its transcription, promoting the migration of T cells from secondary lymphoid organs to peripheral tissues (22). Our results showed that TGP could significantly inhibit the mRNA expression of S1PR1 and KLF2 in MLNs, curbing the excretion of Th17 cells from the intestinal mucosal immune system. CCR6, a critical surface marker of Th17 cells, can guide Th17 cells to migrate to inflammatory sites and secrete cytokines by binding to its ligand CCL20. Meanwhile, inflammatory sites also secrete chemokines such as CXCL10 to recruit Th17 cells. Integrin α4β7, a core molecule for intestinal homing, interacts with its ligand MAdCAM-1, which is predominantly expressed in intestinal LP (24). In addition, CCR9 expressed on T cells specifically binds to CCL25 secreted by intestinal epithelial cells, directing T cell migration to small intestinal mucosa (30). Flow cytometry results showed that TGP could notably reduce the proportion of migratory CCR6^+^ Th17 cells in SMG and intestinal LP, as well as the proportion of CCR9^+^ Th17 and α4β7^+^ Th17 cells in the small intestinal epithelium and LP; RT-qPCR further demonstrated that the mRNA expression levels of the chemokines/adhesion molecules CCL20, CCL25, and MAdCAM-1 were significantly decreased in the SMG, LG, and small intestine of TGP-treated mice. These findings collectively suggest that TGP inhibits the migration of egressed Th17 cells to target tissues and their homing to the intestinal mucosa via the CCR6/CCL20, α4β7/MAdCAM-1, and CCR9/CCL25 axes. At the same time, our immunofluorescence findings showed that TGP markedly decreased the percentage of intestinal mucosa-derived (CFSE-labeled) Th17 cells in the SMG of SS mice, providing direct evidence that TGP suppresses Th17 cell trafficking to the affected target organs (**Figures 6**, **7**). Moreover, in the pathological condition of SS, exocrine glands are attacked by autoimmunity, and glandular cells will be damaged and release autoantigens. These antigens, after being processed by DCs, will activate DCs and affect the differentiation of Th17 cells in exocrine glands. Our results showed that after TGP administration, the proportion of activated DCs in SMG of SS mice was substantially diminished, and the mRNA expression levels of DC effector factors IL-6 and IL-23 in SMG and LG were also significantly decreased, suggesting that TGP could also reduce the differentiation of Th17 cells in exocrine glands by inhibiting the activation of DCs and the secretion of effector factors (**Figure 5**). This aligns with the flow cytometry results, that is, TGP markedly reduced the proportion of Th17 cells in peripheral blood, SMG, and submandibular lymph nodes of SS mice (**Figure 4**).

Finally, we clarified the molecular mechanism of TGP inhibiting the differentiation of Th17 cells in intestinal mucosal immunity through *in vitro* sorting of naive CD4^+^ T cells from MLNs and PPs. The results showed that whether IL-6 and other cytokines were added or co-cultured with activated BMDCs, TGP could inhibit the differentiation of naive CD4^+^ T cells into Th17 cells by reducing the proliferation of CD4^+^ T cells and the expression of key transcription factors RORC, STAT3 and TAZ that regulate Th17 cell differentiation (**Figures 8**, **9**). However, in stark contrast to our *in vivo* observations, our *in vitro* experiments revealed that while LPS robustly promotes the maturation of BMDCs, treatment with TGP and PF does not diminish the proportion of mature DCs or modulate the mRNA expression of IL-6 and other cytokines related to Th17 cell differentiation that are secreted by DCs (**Figure 9**). On the basis of these salient findings, we posit two hypotheses: First, TGP may not exert direct effects on DCs, and its *in vivo* impact on DCs could be an indirect consequence of the intricate *in vivo* environment. Second, TGP only cannot affect the activation of DCs caused by LPS, and subsequent studies can try to replace other stimulants.

In summary, the present study initially establishes the critical role of intestinal mucosal immunity in the pathogenesis and progression of SS. Building on these foundational insights, *in vivo* and *in vitro* studies investigate the molecular mechanism of TGP affecting the differentiation and migration of Th17 cells in intestinal mucosal immunity, revealing the pathogenic mechanism of drugs in alleviating the inflammatory response of exocrine glands and intestinal mucosa in SS. This study not only provides deeper mechanistic insights into the pathogenesis of SS but also offers a robust scientific rationale for understanding the efficacy of poorly absorbed traditional Chinese medicinal compounds like TGP. Nevertheless, this study also has certain limitations. For instance, we did not further explore the effects of TGP on the activation of DCs from other sources or BMDCs induced by stimuli other than LPS. Moreover, due to technical constraints, our experimental design focused solely on two major components of the GALT: PPs and MLNs, without addressing other regions such as the LP. Furthermore, although we focused on demonstrating that TGP suppresses Th17 cell differentiation in the intestinal mucosa of SS mice by reducing CD4^+^ T cell proliferation and the expression of Th17-related transcription factors RORC and STAT3, we did not investigate the upstream mechanisms by which TGP affects these factors. Future research endeavors will continue to explore these areas, overcome these limitations, and expand our understanding of the complex interplay between intestinal mucosal immunity and SS pathogenesis.

## 5 Conclusion

This study demonstrates that the pathogenesis of SS is intricately linked to intestinal mucosal immunity. Meanwhile, TGP exerts evidently inhibitory effects on the proliferation of CD4^+^ T cells, the differentiation and excretion of Th17 cells, along with the activation and migration of DCs within the intestinal mucosal immune system. It can also effectively suppress the migration of differentiated Th17 cells from the intestinal mucosal immune system to the exocrine glands by reducing the expression of CCR6 on Th17 cells and the mRNA expression of chemokines like CCL20 in exocrine glands, and their homing back to the intestinal mucosa by reducing the expression of CCR6, CCR9, and α4β7 on Th17 cells and the mRNA expression of their corresponding ligands CCL20, CCL25, and MAdCAM-1 in the small intestine. Consequently, it decreases the proportion of Th17 cells in peripheral blood and exocrine glands. *In vitro* investigations reveal that although TGP has no effect on DC activation induced by LPS, it notably attenuates the differentiation of naive CD4^+^ T cells in intestinal mucosal immunity into Th17 cells by reducing the proliferation of CD4^+^ T cells and the mRNA expression of Th17-specific transcription factors.

## Data Availability

The raw data supporting the conclusions of this article will be made available by the authors, without undue reservation.
