# Supplementary material for: Total glucosides of paeony inhibits the differentiation and migration of intestinal mucosal immune Th17 cells in Sjogren’s syndrome mice
Source: Front Immunol. 2026 Oct 13;17:1742485. doi: 10.3389/fimmu.2026.1742485 (PMC13710020; doi:10.3389/fimmu.2026.1742485)
Supplement: Supplementary file 1 [file Supplementaryfile1.pdf]

frontiers

The International Society for the Advancement of Cytometry (ISAC) has highlighted the importance of including comprehensive methodological information to ensure data reproducibility and reliability. In line with this, Frontiers in Immunology now requires authors to submit a checklist for manuscripts that involve flow or mass cytometry. This checklist helps standardize the reporting process, improving the quality and transparency of published data. By doing so, we support scientific progress, making it easier for other researchers to replicate and validate experiments.

This form should be submitted with any manuscripts using flow or mass cytometry.

Sample/specimen/material description

- [x] Total blood
- [ ] PBMCs
- [x] Organ digests

Other Peyer's patches, Mesenteric lymph nodes, submandibular lymph nodes, Submandibular glands

Did the samples suffer any treatment before or after incubation with the antibodies?

- [x] Drug Total glucosides of paeony
- [x] Cell permeabilization Fixation/Permeabilization Buffer Set
- [ ] Dye
- [ ] Propidium iodine
- [ ] Not applicable

Other

Instrument and antibodies

Name of the Cytometer BD FACSCelesta Cell Analyzer

**Antibodies and targets**<br>anti-CD4	**Fluorochrome/ Metal**<br>FITC	**Catalog number/Company**<br>11-0042-82/eBioscience
e.g. anti-CD4	FITC	Cat. XXX/ XXX ltd.
anti-CD103	APC	17-1031-80/eBioscience

anti-CD45	BV421	48-0451-80/eBioscience
anti-CD11c	FITC	11-0114-81/eBioscience
anti-MHCII	PE	12-5321-81/eBioscience
anti-CD80	BV605	63-0801-80/eBioscience
anti-CD86	APC	17-0862-81/eBioscience
anti-CCR9	eFluorTM 450	48-1991-82/eBioscience
anti-CCR6	PE	129803/BioLegend
anti-α4β7	PE	12-5887-81/eBioscience
anti-Ki-67	eFluorTM 660	50-5698-80/eBioscience
anti-IL-17A	APC	17-7177-81/eBioscience
anti-RORγt	PE	12-6981-80/eBioscience

Data analyses

1. Name of the software FlowJo_________________________

2. Reference gating strategy in the manuscript or supplementary material

Gating strategy in (eg Figure X) Supplementary Fig. 3
