# Supplementary material for: Total glucosides of paeony inhibits the differentiation and migration of intestinal mucosal immune Th17 cells in Sjogren’s syndrome mice
Source: Front Immunol. 2026 Oct 13;17:1742485. doi: 10.3389/fimmu.2026.1742485 (PMC13710020; doi:10.3389/fimmu.2026.1742485)
Supplement: Supplementary file 2 [file DataSheet1.doc]

***Supplementary Material***

**Supplementary Table 1.** **Gene-specific primers used in the experiment**

| **Genes of interests** | **Primers** |
| --- | --- |
| β-actin | F: CATCCGTAAAGACCTCTATGCCAAC |
|  | R: ATGGTGCCACCGATCCACA |
| IL-17A | F: TTTAACTCCCTTGGCGCAAAA |
|  | R: CTTTCCCTCCGCATTGACAC |
| RORC | F: AACTGGCTTTCCATCATCATCTCTG |
|  | R: GGGAAGGCGGCTTGGACCACGAT |
| STAT3 | F: CAATACCATTGACCTGCCGAT |
|  | R: GAGCGACTCAAACTGCCCT |
| TAZ | F: GAGCAACATGGACGAGATGG |
|  | R: AGGTTAGAAAGGGCTCGCTT |
| IL-6 | F: TAGTCCTTCCTACCCCAATTTCC |
|  | R: TTGGTCCTTAGCCACTCCTTC |
| IL-23 | F: ATGCTGGATTGCAGAGCAGTA |
|  | R: ACGGGGCACATTATTTTTAGTCT |
| IL-1β | F: GCAACTGTTCCTGAACTCAACT |
|  | R: ATCTTTTGGGGTCCGTCAACT |
| TGF-β | F: CTCCCGTGGCTTCTAGTGC |
|  | R: GCCTTAGTTTGGACAGGATCTG |
| CCL20 | F：GCCTCTCGTACATACAGACGC |
|  | R：CCAGTTCTGCTTTGGATCAGC |
| CXCL10 | F：CCAAGTGCTGCCGTCATTTTC |
|  | R：GGCTCGCAGGGATGATTTCAA |
| CCL25 | F：TTACCAGCACAGGATCAAATGG |
|  | R：CGGAAGTAGAATCTCACAGCAC |
| MAdCAM-1 | F：CTGAGCCCTACATCCTGACCT |
|  | R：GCTTCACAGAGTAGCTCCCAG |
| S1PR1 | F：ATGGTGTCCACTAGCATCCC |
|  | R：CGATGTTCAACTTGCCTGTGTAG |
| KLF2 | F：CTCAGCGAGCCTATCTTGCC |
|  | R：CACGTTGTTTAGGTCCTCATCC |

**Supplementary Figure 1: The tissue distribution of PF in SS rats.** Two weeks after TGP (495 mg/kg) administration, the concentrations of PF in the plasma, SMG, LG, spleen, PPs, and MLNs of SS rats were detected by UPLC-MS/MS. All data are mean ± SD, *n* = 3.

**Supplementary Figure 2: TGP reduces the mRNA expression of Th17-related cytokines in intestinal mucosal immunity.** SS mice were treated with vehicle (0.5% CMC-Na), PF (300 mg/kg), or TGP (720, 360 mg/kg) for 6 weeks via intragastric gavage. IL-17A, RORC, STAT3 and TAZ mRNA expression levels in PPs **(a)**, small intestine **(b)**, and MLNs **(c)** of mice were detected by RT-qPCR assay (*n* = 4). All data are mean ± SD. ^⁎^*P* < 0.05, ^⁎⁎^*P* < 0.01, compared to the Model group.

**Supplementary Figure 3: Flow cytometry gating strategies for different cell populations.**
